# A water extract of Samchulkunbi-tang attenuates airway inflammation by inhibiting inos and MMP-9 activities in an ovalbumin-induced murine asthma model

**DOI:** 10.1186/1472-6882-12-257

**Published:** 2012-12-17

**Authors:** Mee Young Lee, In Sik Shin, Hye Sun Lim, Hyeun Kyoo Shin

**Affiliations:** 1 Basic Herbal Medicine Research Group, Korea Institute of Oriental Medicine, 483 Expo-ro, Yusung-gu, Daejeon, 305-811, Republic of Korea

**Keywords:** Samchulkunbi-tang, Asthma, Cytokines, iNOS, MMP-9, Inflammation

## Abstract

**Background:**

In this study, we investigated the effect of Samchulkunbi-tang water extract (SCTE) in an established mouse model of ovalbumin (OVA)-induced allergic asthma. The effects of SCTE on the production of Th1 and Th2 cytokines, eotaxin, and total and OVA-specific immunoglobulin E, inducible nitric oxide synthase expression, and matrix metalloproteinase-9 activity were measured.

**Methods:**

Mice were sensitized on days 0 and 14 with an intraperitoneal injection of 20 μg ovalbumin (OVA) emulsified in 2 mg aluminum hydroxide in 200 μL PBS buffer. On days 21, 22, and 23, mice received an airway exposure to OVA (1%, w/v, in PBS) for 1 h. SCTE was administered orally to mice at doses of 200 and 400 mg/kg per day from days 18 to 23.

**Results:**

SCTE reduced the number of inflammatory cells, cytokines, and chemokines in bronchoalveolar lavage fluids and iNOS expression and MMP-9 activity in mouse lung tissue. Histological studies using hematoxylin & eosin and periodic acid-schiff staining showed that SCTE substantially inhibited OVA-induced inflammatory cell infiltration in lung tissue and goblet cell hyperplasia in the airway. SCTE also reduced IL-4 and IL-13 expression in concanavalin-A-stimulated splenocytes. These results were similar to those obtained with montelukast as a positive control.

**Conclusions:**

Collectively, these results suggest that SCTE may be an effective oral treatment for allergic airway inflammation by virtue of its anti-inflammatory activity.

## Background

Asthma, one of the most prevalent diseases worldwide, is a chronic respiratory disease characterized by heightened airway inflammation, airway hyperresponsiveness, and airflow obstruction in response to specific triggers. The chronic inflammation is associated with airway hyperresponsiveness that leads to recurrent episodes of wheezing, breathlessness, chest tightness, and coughing, particularly at night or in the early morning. These episodes are usually associated with widespread but variable airflow obstruction that is often reversible either spontaneously or with treatment [1]. Eosinophilic inflammation, which has long been considered as important pathogenesis hallmark of asthma, features in many contemporary definitions of asthmatic disease [2]. The mechanism responsible for asthma involves infiltration of eosinophils into the lung, where they preferentially stimulate T-helper 2 (Th2) cell responses by presenting antigens [3]. Therefore, Th2 cells are important primarily in the airways [4], and Th2 cytokines such as interleukin (IL)-4, IL-5, and IL-13 play pivotal roles in the pathophysiology of asthma [5]. IL-33 has recently emerged as a potential therapeutic target in the treatment of asthma [6]. Excessive release of IL-33 from asthmatic bronchial epithelial cells may occur in response to insults from infectious agents, allergens, and pollutants [7] because the chronically inflamed asthmatic epithelium is more susceptible to injury than is normal epithelium.

NO level increases in the airways in animal models of asthma and in patients with asthma [8]. Measurement of exhaled NO has been suggested as helpful for monitoring airway inflammation in asthma, especially in cases of exacerbated asthma [9]. Another important aspect of asthma is that the matrix metalloproteinase-9 (MMP-9) level increases significantly in the bronchoalveolar lavage fluid (BALF), blood, and sputum of people with asthma [10]. MMP-9 belongs to a family of extracellular proteases that are responsible for the degradation of the extracellular matrix during tissue remodeling [11]. Therefore, the control of nitric oxide synthase (NOS) and MMP-9 activities is an important aspect of asthma treatment.

Samchulkunbi-tang (shen zhu jian pi tang in Chinese) is a herbal formula that is used widely in Korean traditional medicine in the treatment of chronic gastritis, gastric ulcers, and gastroptosis. Several researchers have reported that Samchulkunbi-tang has pharmacological activities in processes such as immune regulation [12] and gastroprotection [13]. Ginseng radix, one of the constituents of Samchulkunbi-tang, has been used to prevent various diseases including diabetes, cancer, allergy, and hypertension [14] and to treat inflammation [15]. Atractylodis rhizoma alba, another constituent of Samchulkunbi-tang, has been reported to inhibit melanin biosynthesis [16]. However, to our knowledge, there are no reports on the antiasthmatic activity of Samchulkunbi-tang*.* Patients with chronic asthma who take antiasthmatic drugs (including corticosteroids) for a long and indefinite time may develop serious side effects [17]. The present study was designed to determine if SCTE has anti-inflammatory and antiasthmatic effects in a mouse model of OVA-induced airway inflammation.

## Methods

### Preparation of SCTE

Samchulkunbi-tang was prepared according to a transitional herbal formula listed in Table 1. The chopped herbs were combined in the listed ratios and extracted in distilled water at 100°C for 2 h. The yield of lyophilized extract from starting crude materials was about 24.5%. The dried extract was dissolved in PBS.

**Table 1:** Composition of samchulkunbi-tang water extract (SCTE)

**Latin name**	**Amount(g)**
Ginseng Radix	3.750
Atractylodis Rhizoma Alba	3.750
Hoelen	3.750
Maglonia Cortes	3.750
Aurantii Nobilis pericarpium	3.750
Crataegii Fructus	3.750
Ponciri Fructus	3.000
Paeoniae Radix	3.000
Amomi Fructus	1.875
Massa Medicata Fementata	1.875
Hor dei fructus Ger minitus	1.875
Glyeyrhizae Radix	1.875
Zingiberis Rhizoma	3.750
Zizyphi Jujubae Fructus	3.750
Total	43.500

### Experimental protocol

Seven-week-old female BALB/c pathogen-free mice were purchased from Orient Co. Ltd (Seoul, Korea) and maintained in an animal facility under standard laboratory conditions for one week before the experiments. Animals were provided water and standard chow *ad libitum*. All experimental procedures were carried out in accordance with the NIH Guidelines for the Care and Use of Laboratory Animals and were approved by Korea Institute of Oriental Medicine Institutional Animal Care and Use Committee. The animals were cared for in accordance with the dictates of the National Animal Welfare Law of Korea. The modified protocols for mice sensitization and challenge were used as described previously [18].

### Administration of SCTE

**Figure 1:**
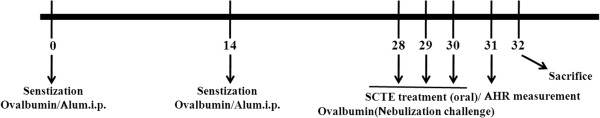
Mouse model of airway inflammation and effects of SCTE.

SCTE (200 mg/kg or 400 mg/kg) was administered orally once daily on days 28–30. Negative control (NC) and positive control mice were treated orally with PBS and montelukast (30 mg/kg, Sigma, St Louis, MO, USA), respectively, once daily on days 28–30. Animals were sacrificed 48 h after the last challenge (i.e., on day 32) to characterize the effects of SCTE. A schematic diagram of the treatment schedule is shown in Figure 1.

### Measurement of total cell, eosinophil, lymphocyte, neutrophil, and macrophage cell counts in BALF

Differential cell counting was performed as described previously [18].

### Measurement of cytokine and chemokine levels in BALF

Levels of IL-4, IL-13, IL-33, tumor necrosis factor-α (TNF-α), and eotaxin in BALF were measured using enzyme-linked immunosorbent assay (ELISA) kits according to the manufacturer’s instructions (BioSource International, Camarillo, CA, USA) as described previously [18]. The ranges of detection for IL-4 and IL-5 are 0 to 1000 pg/mL and 0 to 500 pg/mL, respectively.

### Measurement of total and OVA-specific immunoglobulin E (IgE) levels in plasma

Serum was collected via centrifugation (200 × *g*, 10 min) and stored at −70°C. Total and OVA-specific IgE levels were measured using ELISAs as described previously [18].

### Histopathology

For histological examination, before the lungs were removed, the left lungs were filled intratracheally with a fixative (0.8% formalin, 4% acetic acid) using a ligature around the trachea. The tissues were embedded in paraffin, sectioned at 4 μm thickness, and stained with hematoxylin and eosin (H&E) (both from Sigma) and periodic acid-Schiff (PAS) solution (IMEB Inc., San Marcos, CA, USA) to assess mucus production. Tissues were mounted and coverslips were attached using mounting medium (Invitrogen, CA, USA). The degree of cell infiltration in the airway was scored in a double-blind screen by two independent investigators. The peri-bronchiole and peri-vascular inflammation was evaluated using a score of 0–5 as described previously [19]. For each mouse, five airway sections that were randomly distributed through the left lung were analyzed, and their average scores were calculated. Quantitative analysis of mucus production was performed using an image analyzer (Leica Microsystem Imaging Solutions Ltd., Cambridge, UK).

### Measurement of MMP-9 level in lung tissue

Zymography in lung tissue was performed as described previously [19] with some modifications. Lung tissues were homogenized (1/10 w/v) in tissue lysis/extraction reagent plus protease inhibitor (Sigma-Aldrich) to obtain extracts of lung tissues. After centrifugation (12,000 g, 4°C, 10 min), the protein concentration in the supernatants was determined using a protein assay reagent (Bio-Rad Laboratories) according to the manufacturer’s instructions, and equal amounts of total protein were loaded for gelatin zymography (60 μg/lane).

### Western blotting

Equal amounts of total lung protein (30 μg) were heated at 100°C for 5 min, loaded onto 8% SDS-PAGE gels, and separated by electrophoresis, after which the bands were transferred to a nitrocellulose membrane (at 100 V for 2 h). The membranes were blocked for 1 h with Tris-buffered saline containing 0.05% Tween 20 (TBST) plus 5% skim milk and were incubated with anti-inducible NOS (iNOS, 1:1000 dilution), anti-NF-κB p65, anti-β-actin (1:1000 dilution), and anti-MMP-9 (1:1000 dilution) overnight at 4°C. The membranes were washed three times with TBST and then incubated with a 1:10,000 dilution of horseradish peroxidase-conjugated secondary antibody for 1 h at room temperature. The membranes were washed three times with TBST and then developed using an enhanced chemiluminescence kit.

### Preparation and treatment of splenocyte suspensions

Spleens from BALB/c mice were removed aseptically, and single-cell suspensions were generated by passing the cells twice through a needle in RPMI 1640 medium containing 10% (v/v) FBS, 25 mM HEPES, 2 mM glutamine, 100 U/mL penicillin, and 100 mg/mL streptomycin (GibcoBRL, NY, USA). The red blood cells were lysed in lysis buffer (Sigma) at 37°C for 10 min. The separated splenocytes were washed with PBS and cultured in 100 mm dishes for 4 h. The splenocytes were plated into 96-well plates at a density of 1 × 10^6^ cells/mL and treated with different concentrations of *p*-hydroxycinnamic acid methyl ester for 1 h, followed by treatment with concanavalin A (ConA; 1 μg/mL) for a further three days. The IL-4 and IL-13 levels in the culture supernatants were measured with ELISA kits for murine cytokines (BioSource International) according to the manufacturer’s instructions.

### Statistical analysis

The data are expressed as mean ± standard deviation. Statistical comparisons were performed using one-way analysis of variance, with significance set at *P <* 0.05 or *P <* 0.01.

## Results

### Effects of SCTE on cell numbers in BALF

**Figure 2:**
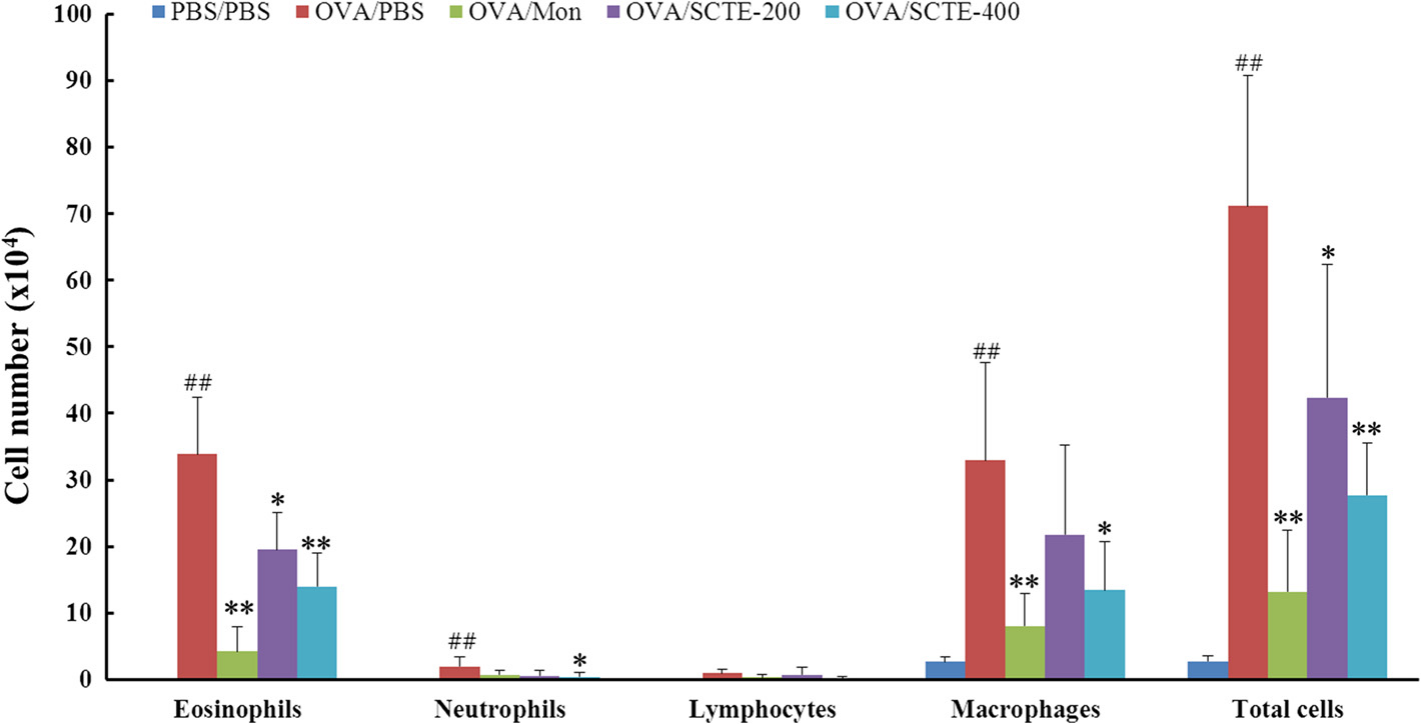
**Effects of SCTE on the recruitment of inflammatory cells to bronchoalveolar lavage fluid (BALF) of mice 48 h after the final ovalbumin (OVA) challenge.** Cells were isolated by centrifugation and stained with Diff-Quik® Stain reagent. Cell numbers were determined by counting within at least five squares of a hemocytometer using a light microscope. Dead cells, stained with Trypan blue, were excluded from the total cell count. PBS/PBS, PBS-sensitized/challenged, negative control (NC, PBS only administration); OVA/PBS, OVA-sensitized/challenged mice (PBS only administration); OVA/mon, OVA-sensitized/challenged mice (montelukast 30 mg/kg); OVA/SCTE-200, OVA-sensitized/challenged mice (SCTE 200 mg/kg); OVA/SCTE-400, OVA-sensitized/challenged mice (SCTE 400 mg/kg). SCTE or montelukast was given 1 h before the challenge. Significantly different from PBS/PBS, ^##^*P <* 0.01; significantly different from OVA, ^*^P < 0.05, ^**^P < 0.01.

Infiltration of eosinophils in the airway causes abnormal production of inflammatory proteins and cytokines, such as IL-4, IL-5, IL-6, and IL-13. We investigated the effects of SCTE on various cell types present in BALF. As shown Figure 2, the numbers of total cells, macrophages, and eosinophils in BALF decreased significantly in a dose-dependent manner after SCTE treatment. The positive control also showed a significant decrease in total cell number in BALF after SCTE treatment (Figure 2).

### Effects of SCTE on Th2-type cytokine and chemokine levels in BALF

**Figure 3:**
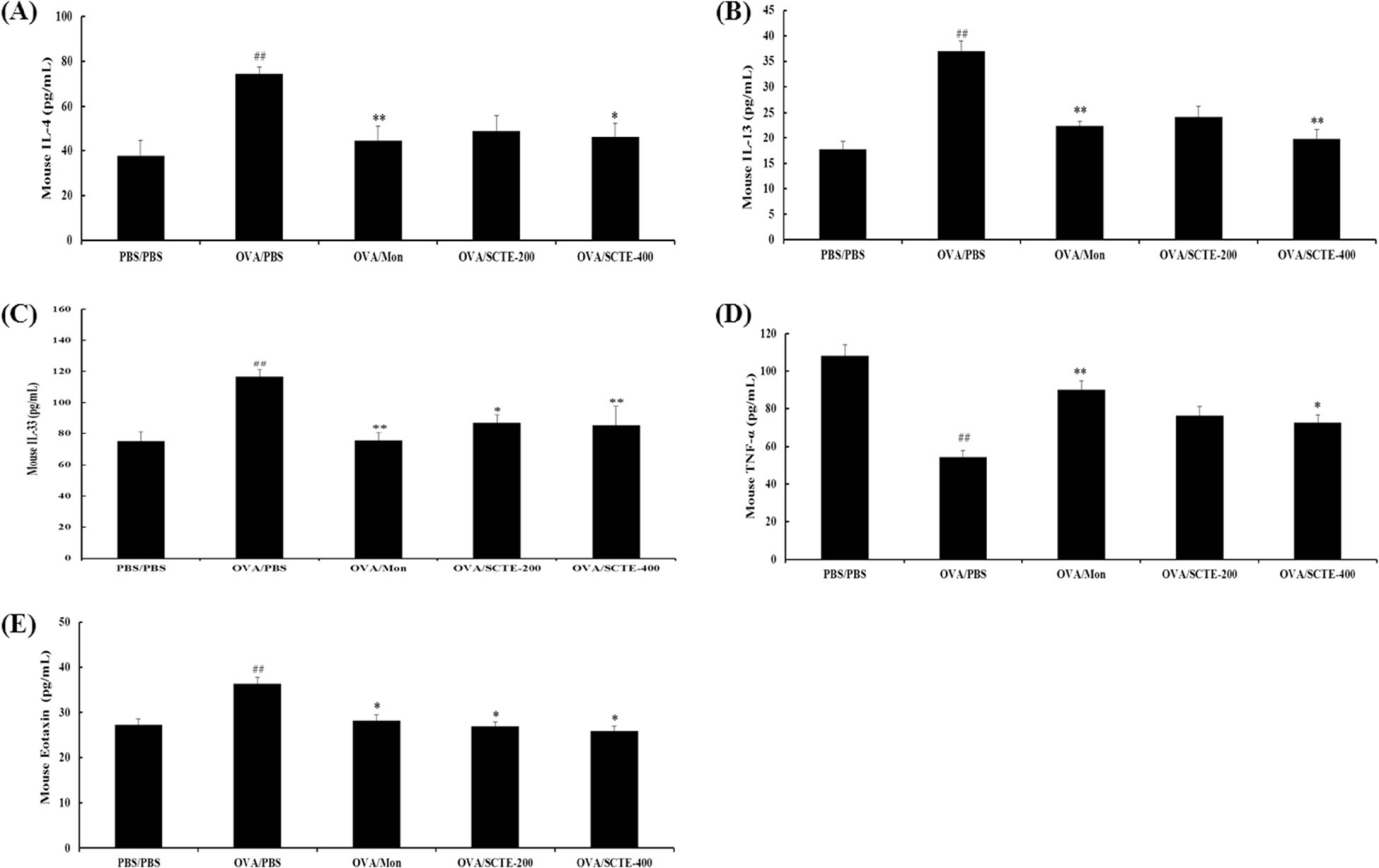
**Effects of SCTE on cytokine and chemokine levels in BALF.** BALF was collected from mice 48 h after the last OVA challenge. Individual samples were analyzed using ELISA. (**A**) IL-4; (**B**) IL-13; (**C**) IL-33, (**D**) TNF-α, (**E**) eotaxin. PBS/PBS, PBS-sensitized/challenged, negative control (PBS only); OVA/PBS, OVA-sensitized/challenged mice (PBS only); OVA/mon, OVA-sensitized/challenged mice (montelukast 30 mg/kg); OVA/SCTE-200, OVA-sensitized/challenged mice (SCTE 200 mg/kg); OVA/SCTE-400, OVA-sensitized/challenged mice (SCTE 400 mg/kg). SCTE or montelukast was given 1 h before the challenge. Significantly different from PBS/PBS, ^##^*P <* 0.01; significantly different from OVA, ^*^P < 0.05, ^**^P < 0.01.

Because SCTE reduced the number of inflammatory cells in BALF, we investigated the effects of SCTE on Th2-type cytokines by measuring the levels of IL-4, IL-13, IL-33, and TNF-α. As shown in Figure 3A-D, the levels of these cytokines increased in the OVA-sensitized/challenged group and decreased in the SCTE-treated group compared with the NC group. Because eotaxin level is associated with eosinophilia in BALF, we measured the eotaxin levels in BALF. Similar to the levels of Th2-type cytokines, eotaxin level increased in the OVA-sensitized/challenged group and decreased in a dose-dependent manner in the SCTE-treated group (Figure 3E).

### Effects of SCTE on total and OVA-specific IgE levels

**Figure 4:**
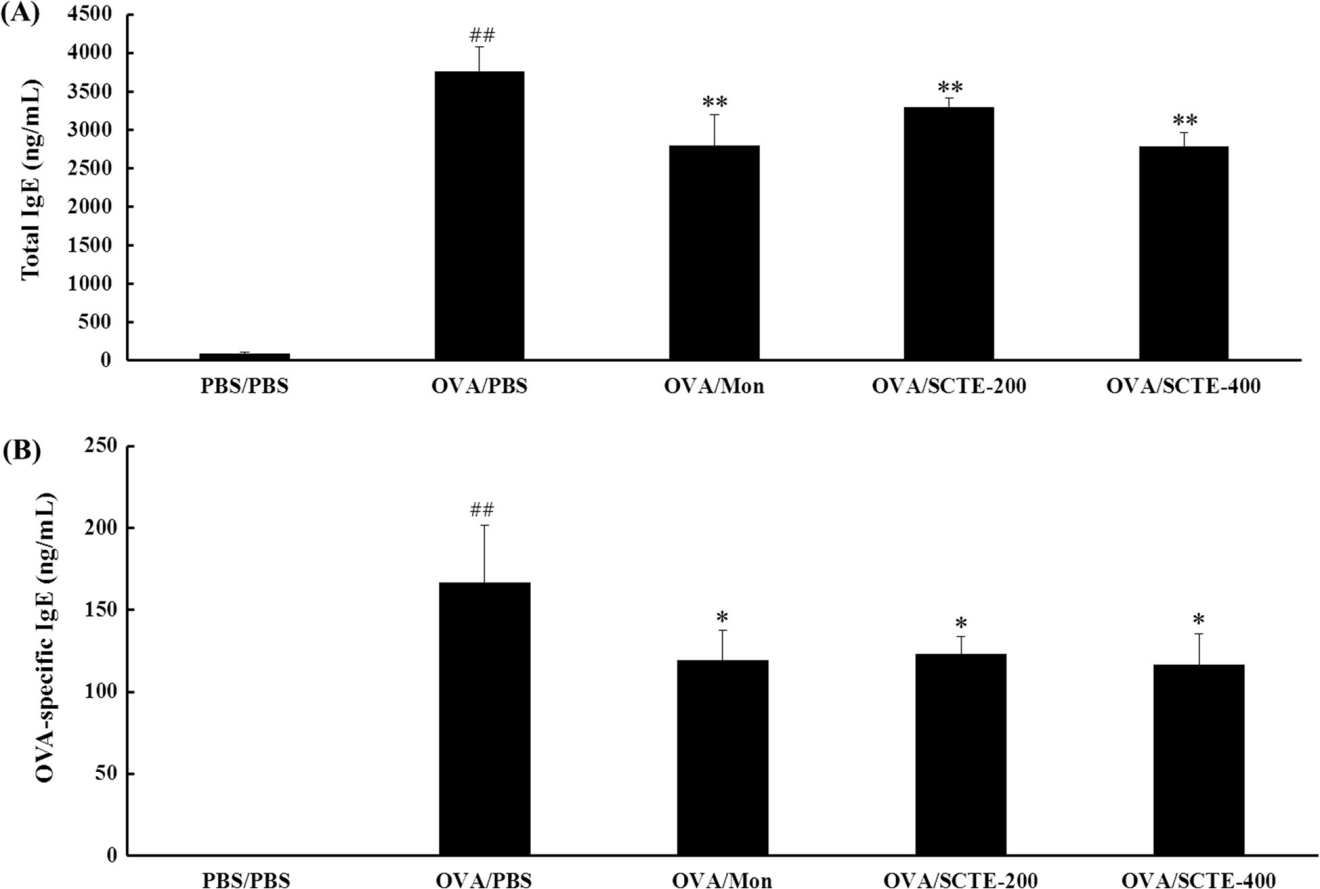
**Effects of SCTE on the levels of total and OVA-specific IgE in plasma.** Plasma was collected from mice 48 h after the final OVA challenge. Each sample was analyzed using ELISA. (**A**) Total IgE level, (**B**) OVA-specific IgE level. PBS/PBS, PBS-sensitized/challenged, negative control (PBS only); OVA/PBS, OVA-sensitized/challenged mice, (PBS only); OVA/mon, OVA-sensitized/challenged mice (montelukast 30 mg/kg); OVA/SCTE-200, OVA-sensitized/challenged mice (SCTE 200 mg/kg); OVA/SCTE-400, OVA-sensitized/challenged mice (SCTE 400 mg/kg). SCTE or montelukast was given 1 h before the challenge. Significantly different from PBS/PBS, ^##^*P <* 0.01; significantly different from OVA, ^*^P < 0.05, ^**^P < 0.01.

Systemic changes observed in the mouse model were examined further by measuring the serum concentrations of total and OVA-specific IgE levels in plasma. The OVA-specific IgE concentration increased in asthmatic mice but was undetectable in nonasthmatic control mice. By contrast, the total and OVA-specific IgE concentrations were lower in the SCTE-treated mice compared with the OVA-induced asthmatic mice (Figure 4A and B).

### Effects of SCTE on airway inflammatory cell recruitment and mucus production in lung tissue

**Figure 5:**
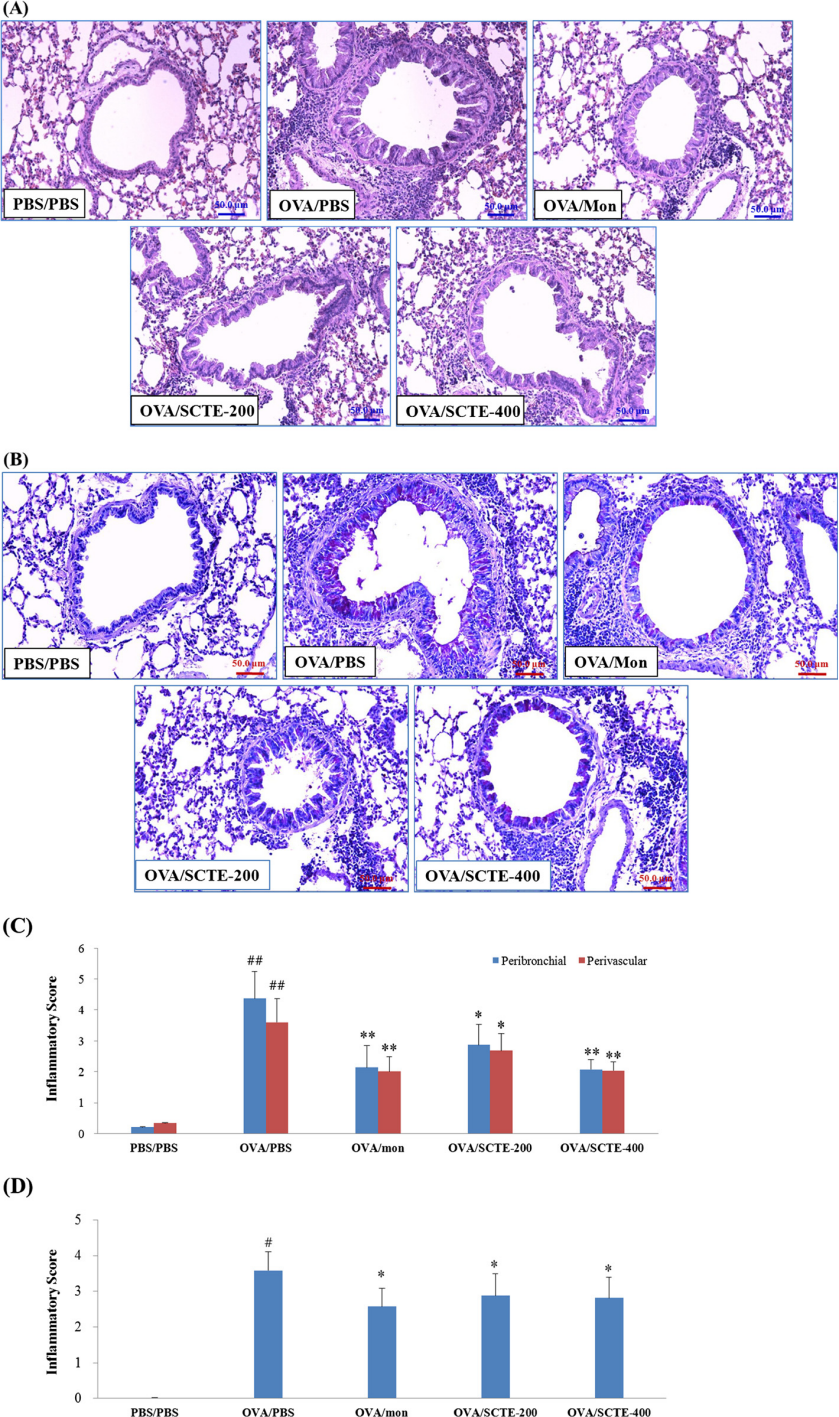
**Effects of SCTE on the recruitment of leukocytes (A), mucus production (B), scoring of the extent of inflammation *via *quantitative analysis of inflammatory cell infiltration (C), scoring of mucus production (D) in lung tissue.** Histological examination of lung tissues was performed 48 h after the final OVA challenge. Lung tissues were fixed, sectioned at 4 μm thickness, and stained with H&E (**A**) solution or PAS (**B**). PBS/PBS, PBS-sensitized/challenged, negative control (PBS only); OVA/PBS, OVA-sensitized/challenged mice, (PBS only); OVA/mon, OVA-sensitized/challenged mice (montelukast 30 mg/kg); OVA/SCTE-200, OVA-sensitized/challenged mice (SCTE 200 mg/kg); OVA/SCTE-400, OVA-sensitized/challenged mice (SCTE 400 mg/kg). SCTE or montelukast was given 1 h before the challenge. Significantly different from PBS/PBS, ^##^*P <* 0.01; significantly different from OVA, ^*^P < 0.05, ^**^P < 0.01.

Lung inflammation is a characteristic hallmark of the allergic response to an allergen. In view of the finding that SCTE inhibited inflammatory cell recruitment into BALF, we examined its antiasthmatic effects via microscopic examination of lung tissue. The extent and the anatomical location of the leukocyte infiltrates were determined by examining H&E-stained tissue obtained from mice 48 h after the final allergen challenge. Tissue from the OVA-sensitized/challenged group showed widespread peribronchiolar and perivascular inflammation, comprising primarily eosinophils. Tissue from the mice given SCTE had substantially fewer eosinophils and macrophages in the peribronchial regions and airspaces compared with the NC group (Figure 5 A and C).

Although respiratory mucus protects the lower airways from dehydration and damage, excessive secretion by hyperplastic goblet cells contributes to the morbidity and mortality of many respiratory diseases, including asthma. To determine whether SCTE suppressed mucus overproduction induced by goblet cell hyperplasia, lung sections were stained with PAS. In OVA-sensitized/challenged mice, mucus overproduction was observed clearly as a violet color in the bronchial airways compared with that observed in the PBS/PBS group. The extent of mucus staining was markedly diminished in a dose-dependent manner in OVA-induced mice treated with SCTE (Figure 5 B and D).

### Effects of SCTE on iNOS and NF-κB p65 levels in lung tissue

**Figure 6:**
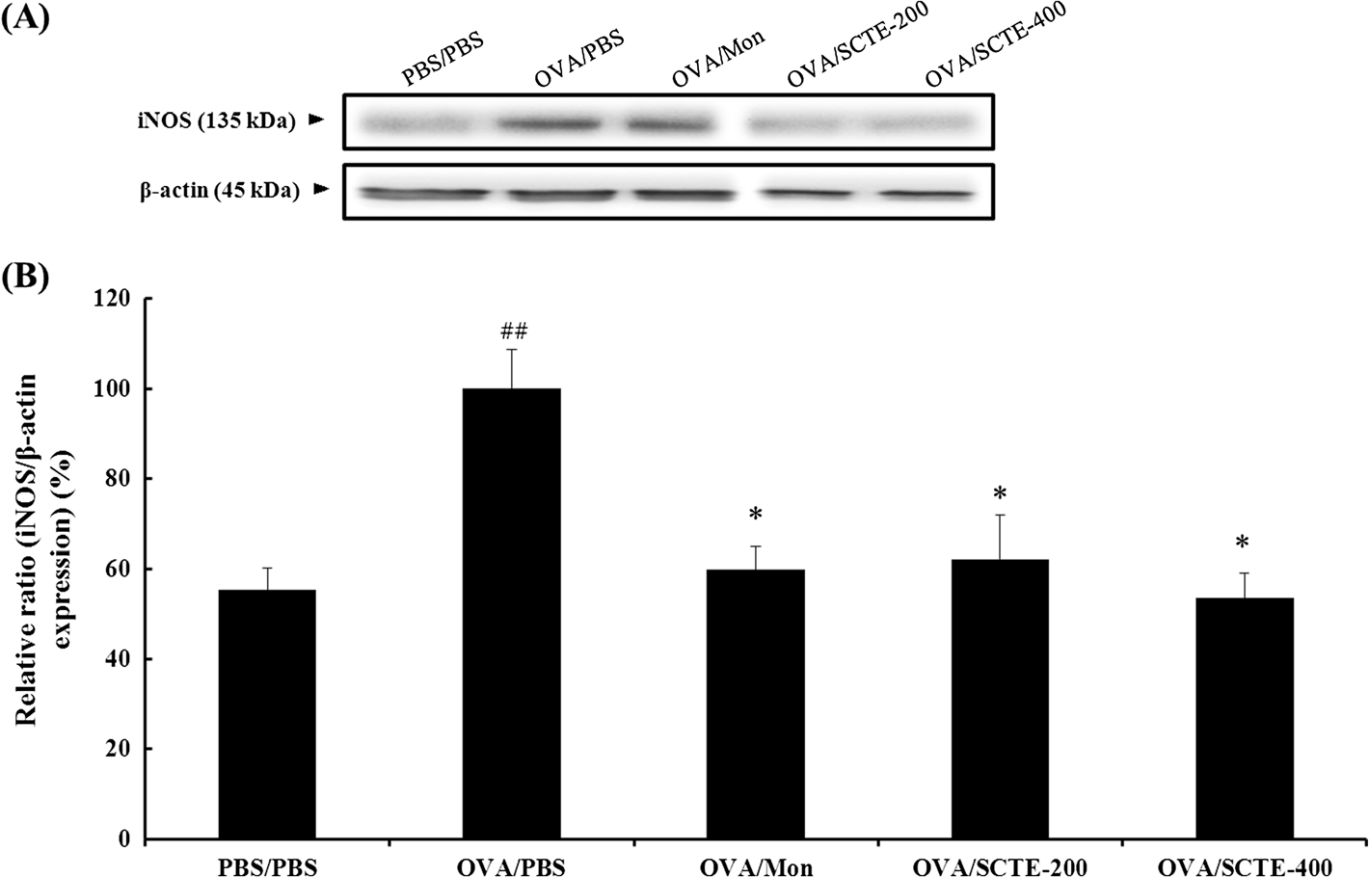
**Effect of SCTE on iNOS expression in lung tissue.** iNOS protein levels were measured 48 h after the last challenge; PBS/PBS, PBS-sensitized/challenged, negative control (PBS only); OVA/PBS, OVA-sensitized/challenged mice, (PBS only); OVA/mon, OVA-sensitized/challenged mice (montelukast 30 mg/kg); OVA/SCTE-200, OVA-sensitized/challenged mice (SCTE 200 mg/kg); OVA/SCTE-400, OVA-sensitized/challenged mice (SCTE 400 mg/kg). SCTE or montelukast was given 1 h before the challenge.

**Figure 7:**
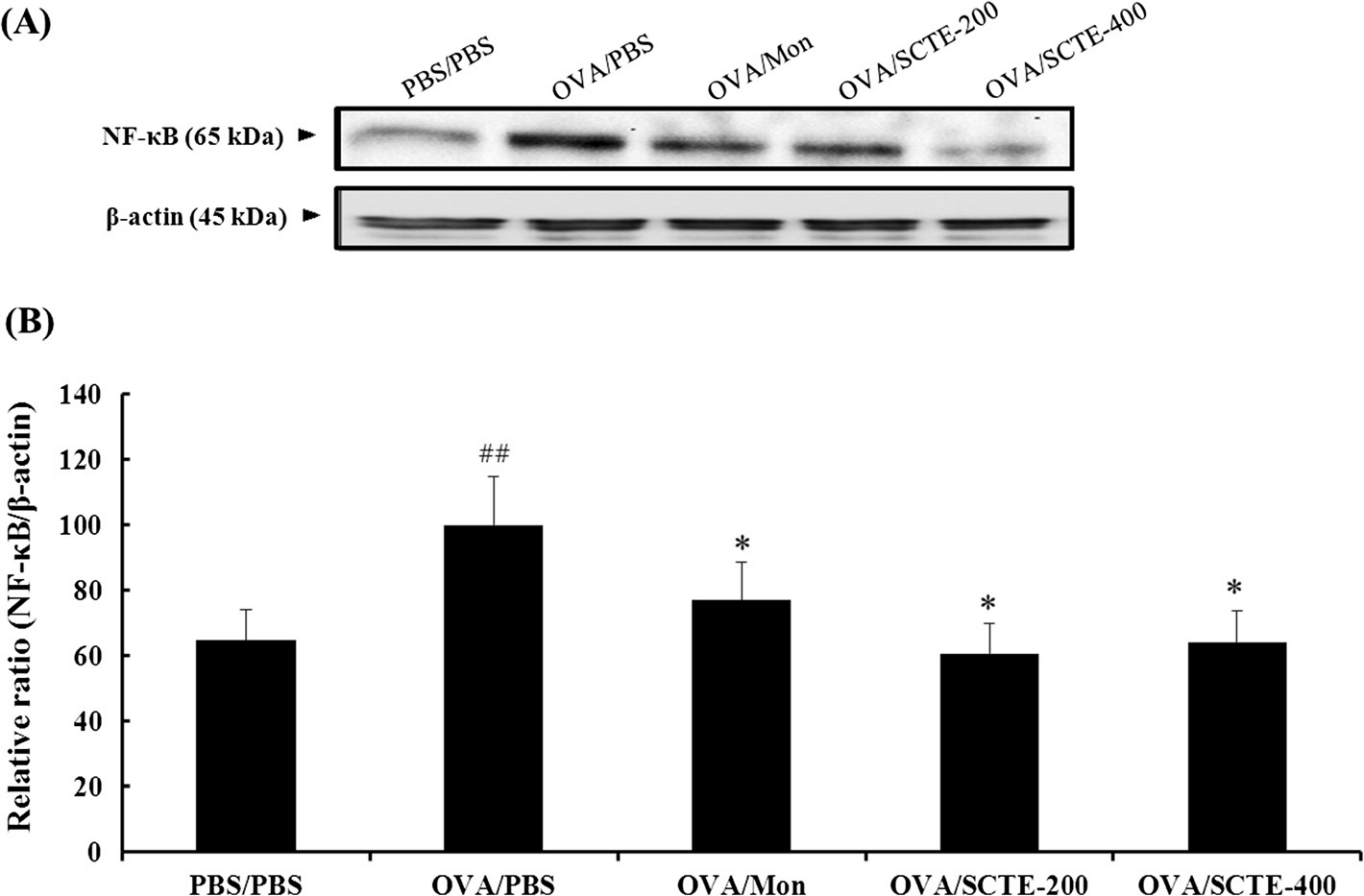
**Effect of SCTE on NF-κB activity in lung tissue.** NF-κB p65 protein level was measured 48 h after the last challenge; PBS/PBS, PBS-sensitized/challenged, negative control (PBS only); OVA/PBS, OVA-sensitized/challenged mice, (PBS only); OVA/mon, OVA-sensitized/challenged mice (montelukast 30 mg/kg); OVA/SCTE-200, OVA-sensitized/challenged mice (SCTE 200 mg/kg); OVA/SCTE-400, OVA-sensitized/challenged mice (SCTE 400 mg/kg). SCTE or montelukast was given 1 h before the challenge.

During OVA-induced allergic airway inflammation, the concentration of iNOS and NF-κB p65 in nuclear protein extracts from lung tissues was increased significantly in OVA-sensitized/challenged mice compared with the NC group. By contrast, the iNOS (Figure 6A and B) and NF-κB p65 (Figure. 7A and B).level was significantly lower in the SCTE-treated group compare to OVA-induced group

### Effects of SCTE on MMP-9 activity in lung tissue

**Figure 8:**
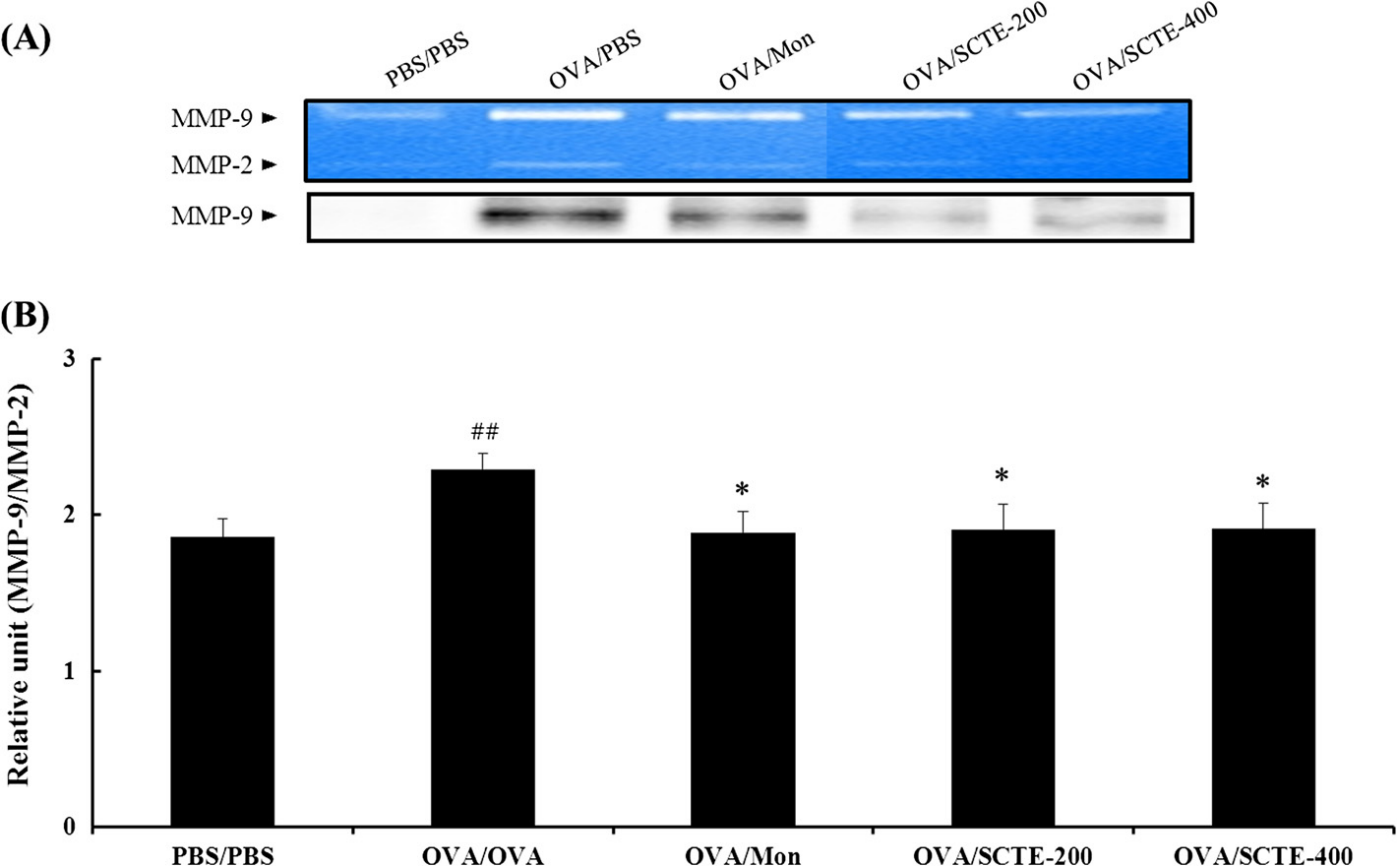
**Effects of SCTE on MMP-9 activity and protein expression in lung tissues of mice.** The protein was loaded for gelatin zymography (60 μg/lane). SDS-PAGE zymography was performed according to previous method [19]. (**A**) MMP-9 activity and protein expression. (**B**) Densitometry of MMP-9. PBS/PBS, PBS-sensitized/challenged, negative control (PBS only); OVA/PBS, OVA-sensitized/challenged mice (PBS only); OVA/mon, OVA-sensitized/challenged mice (montelukast 30 mg/kg); OVA/SCTE-200, OVA-sensitized/challenged mice (SCTE 200 mg/kg); OVA/SCTE-400, OVA-sensitized/challenged mice (SCTE 400 mg/kg). SCTE or montelukast was given 1 h before the challenge. Significantly different from PBS/PBS, ^#^*P <* 0.05, ^##^*P <* 0.01; significantly different from OVA, ^*^P < 0.05, ^**^P < 0.01.

Zymography showed that MMP-9 activity increased in OVA-induced mice but decreased in SCTE-treated OVA-induced mice compared with NC mice. The reduction in MMP-9 activity was consistent with the expression of MMP-9 protein in lung tissue. MMP-9 expression increased in control OVA-induced mice but decreased markedly in SCTE-treated mice (Figure 8A and B).

### Effects of SCTE on Th2-type cytokine production in splenocytes

**Figure 9:**
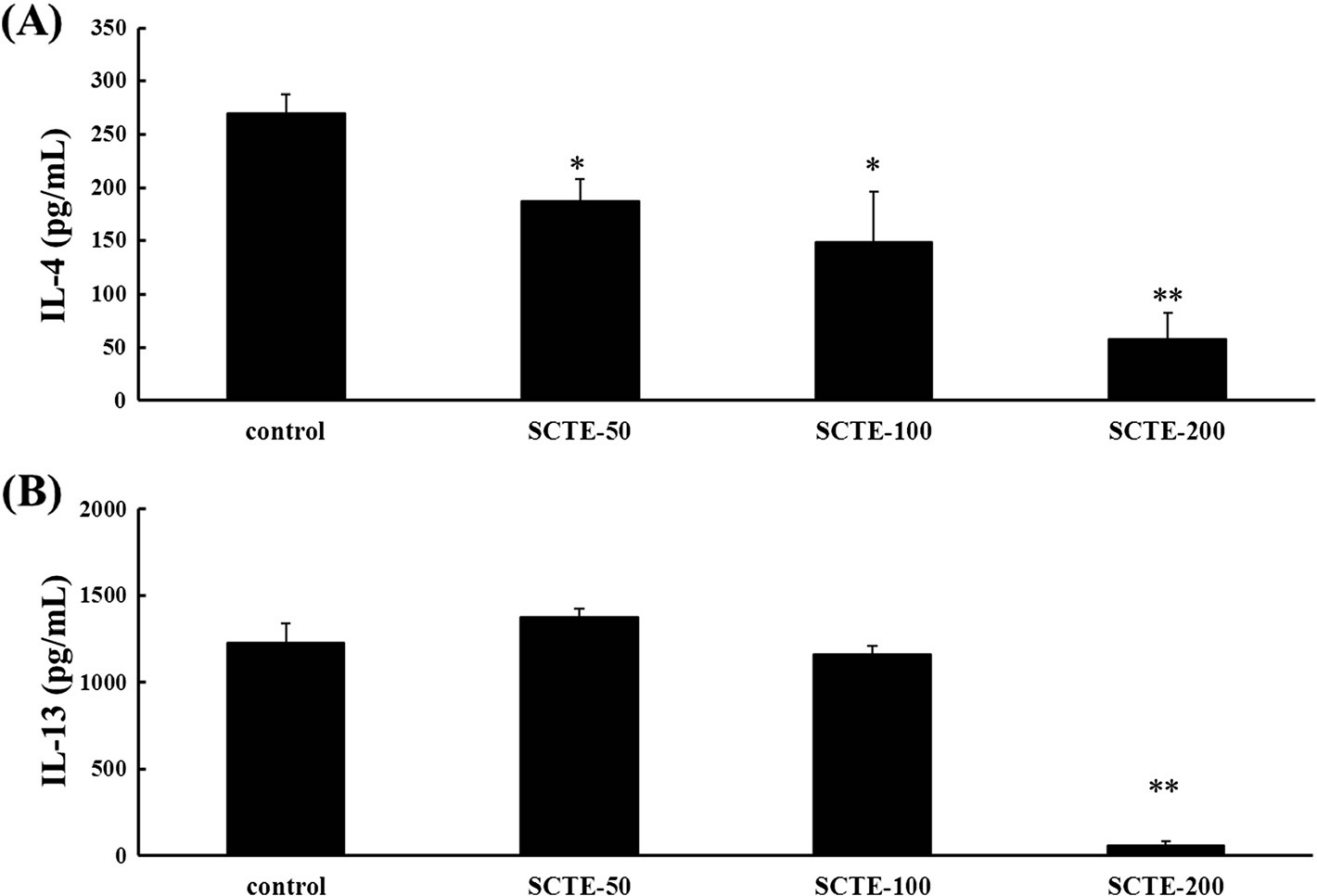
**Effects of SCTE on IL-4 and IL-5 levels in splenocytes.** (**A**) IL-4 level; (**B**) IL-5 level. Control, Con-A-stimulated cells, negative control (PBS only); SCTE-50, Con-A stimulated + SCTE 50 μg/mL; SCTE-100, Con-A stimulated + SCTE 100 μg/mL; SCTE-200, Con-A stimulated + SCTE 200 μg/mL. Significantly different from control, ^*^P < 0.05, ^**^P < 0.01.

We also examined the effects of SCTE on the production of Th2-type cytokines (IL-4 and IL-13) by splenocytes. Treatment with ConA (1 μg/mL) increased IL-4 and IL-13 production markedly in splenocytes. ConA-stimulated IL-4 secretion by splenocytes was inhibited by treatment with various concentrations of SCTE (50, 100, and 200 μg/mL) compared with the control. IL-13 level decreased only at an SCTE dose of 200 μg/mL (Figure 9).

## Discussion

Our results show clearly that SCTE significantly modulated the pulmonary environment of Th1- (TNF-α) and Th2-type cytokines (IL-4, IL-13, and IL-33) and chemokines (eotaxin) in BALF, and inhibited iNOS expression and MMP-9 activity in the mouse lung tissue compared with the effects in OVA-induced mice. SCTE decreased the total and OVA-specific IgE level in plasma. In lung histopathological studies using H&E and PAS staining, SCTE inhibited inflammatory cell infiltration and mucus hypersecretion compared with the effects in OVA-challenged mice. SCTE also reduced IL-4 and IL-13 expression in Con-A-stimulated splenocytes.

Th2-type cytokines such as IL-4, IL-5, and IL-13 play important roles in the development of allergic asthmatic responses in humans [20]. SCTE treatment reduced the number of eosinophils in BALF and in the lung tissue surrounding the airways, and decreased the extent of goblet cell hyperplasia compared with untreated mice. However, there was little change in the numbers of other leukocytes such as neutrophils, lymphocytes, and macrophages. It is possible that the reduction in eosinophil numbers observed in our study reflects a decrease in IL-5-dependent eosinophil expansion. IL-5 plays an important role in the differentiation, maturation, and survival of eosinophils, which lead to an increased number of these cells in the airways subsequent to activation. A previous study has shown that eosinophilic inflammation does not develop in the absence of IL-5 or its signaling in the airways of OVA-sensitized/challenged mice [21].

We found that SCTE reduced the production of IL-4, IL-5, and IL-13. IL-4 promotes the differentiation and proliferation of Th2-type T cells, and the switching of B cells to produce IgG1 and IgE. Blocking of IL-4 by monoclonal antibodies decreases IgE level and airway eosinophilia in allergic mice [22]. Therefore, suppression of IL-4 may also contribute to decreasing lung eosinophilia. Increased immunoreactive IL-33 level has a variety of effects on inflammatory cells. IL-33 is present in the peripheral blood [23] and in BALF of asthmatic patients whose bronchial epithelium produces this cytokine at high levels [24]. IL-33 drives production of proinflammatory and Th2 cytokines by mast cells and Th2 lymphocytes [25,26], induces chemotaxis of Th2 cells [27], promotes eosinophil and basophil adhesion, and increases eosinophil survival and basophil migration [28]. In the present study, IL-33 reduction by SCTE may help decrease lung and BALF eosinophil numbers. Th2 cytokines, especially IL-13, are central mediators of asthma, and IL-13 potently induces goblet cell metaplasia by human airway epithelial cells [29]. Therefore, in the present study, the decrease of goblet hyperplasia may reflect less IL-13 production compared with OVA-induced mice.

TNF-α is also an important chemoattractant for the recruitment of eosinophils into the lungs [30] and is a potent modulator of the immune and inflammatory responses. Inflammatory cells contribute to the generation of Th2 cytokines (IL-4, IL-5, and IL-13), chemokines (eotaxin and RANTES), and TNF-α, whose levels increase in the asthmatic lung [31]. In our experiments, SCTE treatment reduced the levels of IL-4, IL-5, IL-13, TNF-α, and eotaxin; these findings are consistent with the change in inflammatory cell count in BALF. To identify the possible protective mechanism underlying the activity of SCTE in airway inflammation, we used gelatin zymography to evaluate the activity of MMP-9 and Western blotting to evaluate the expression of MMP-9 protein in lung tissue. We were interested in the relationship between MMP-9 expression and infiltration of inflammatory cells in lungs of the OVA-challenged mice. SCTE-treated OVA-induced mice showed reduced activity and protein expression of MMP-9 in lung tissue compared with control OVA-challenged mice. These results are consistent with the observed changes in cytokines. The dose-dependent changes are also consistent with those shown in an *in vivo* experiment in rats [32].

Excessive NO may recruit eosinophils into the airway and shift the balance toward Th2 cells, thus exacerbating airway inflammation [33]. iNOS produces high amounts of NO. The present results from our OVA-induced asthma model showing decreased production of iNOS in lung tissues, increased inflammatory cytokine levels, and recruitment of eosinophils to the lung airways are consistent with the study by Nathan. In our experiments, SCTE significantly reduced goblet cell hyperplasia and mucus production in the OVA-induced murine asthmatic animals. Eosinophils infiltrating into the airway also increase mucus secretion of epithelial goblet cells [34]. These results suggest that mucus hypersecretion is attenuated by the ability of SCTE to limit cytokine production and eosinophilia, and that SCTE can inhibit the development of the allergic status in the OVA-induced asthma model.

## Conclusion

In summary, administration of SCTE in this mouse asthma model significantly decreased the number of eosinophils in BALF and lung tissue, and reduced IL-4 IL-5, IL-13, TNF-α, and eotaxin production in BALF and total IgE and OVA-specific IgE levels in plasma after OVA challenge. Administration of montelukast was induced anti-inflammatory effects such as the reduction in the numbers of eosinophils and macrophage into the BALF, inflammatory cells infiltration in the lung tissue and levels of cytokines and IgE in this study. These findings suggest that SCTE may effectively inhibit the progression of airway inflammation of allergic asthma. The anti-inflammatory effects of SCTE were mediated partially by downregulation of MMP-9 and reduction in iNOS expression.

## Competing interests

No competing financial interests exist.

## Authors’ contributions

MYL and HKS participated in the design of the study data analyses and manuscript preparation. ISS and HSL conducted the assays and analyses. All authors read and approved the final manuscript.

## Pre-publication history

The pre-publication history for this paper can be accessed here:

http://www.biomedcentral.com/1472-6882/12/257/prepub
